# Risk factors affecting the mortality of HIV-infected patients with pulmonary tuberculosis in the cART era: a retrospective cohort study in China

**DOI:** 10.1186/s40249-018-0405-8

**Published:** 2018-03-24

**Authors:** Yong-Jia Ji, Pei-Pei Liang, Jia-Yin Shen, Jian-Jun Sun, Jun-Yang Yang, Jun Chen, Tang-Kai Qi, Zhen-Yan Wang, Wei Song, Yang Tang, Li Liu, Ren-Fang Zhang, Yin-Zhong Shen, Hong-Zhou Lu

**Affiliations:** 1 Department of Infectious Disease, Shanghai Public Health Clinical Center, Fudan University, No. 2901 Cao Lang Rd, Jinshan District, Shanghai, 201508 China; 2 Department of Infectious Disease, Huashan Hospital Affiliated to Fudan University, Shanghai, 200040 China; 3 Department of Internal Medicine, Shanghai Medical College, Fudan University, Shanghai, 200032 China

**Keywords:** HIV infections, Acquired immunodeficiency syndrome, Pulmonary tuberculosis, Survival analysis

## Abstract

**Background:**

Tuberculosis infection still places a great burden on HIV-infected individuals in China and other developing countries. Knowledge of the survival of HIV-infected patients with pulmonary tuberculosis (PTB) would provide important insights for the clinical management of this population, which remains to be well described in current China.

**Methods:**

HIV-infected patients with PTB admitted to Shanghai Public Health Clinical Center from January 2011 to December 2015 were retrospectively enrolled. In this cohort, the survival prognosis was estimated by the Kaplan-Meier method, while univariate and multivariate Cox proportional hazards models were used to determine the risk factors affecting mortality.

**Results:**

After reviewing 4914 admitted patients with HIV infection, 359 PTB cases were identified. At the time of PTB diagnosis, the patients’ median CD4^+^ T cell count was 51 /mm^3^ (IQR: 23–116), and 27.30% of patients (98/359) were on combination antiretroviral therapy (cART). For the 333 cases included in the survival analysis, the overall mortality was 15.92% (53/333) during a median 27-month follow-up. The risk factors, including age older than 60 years (HR: 3.18; 95% *CI*: 1.66–6.10), complication with bacterial pneumonia (HR: 2.64; 95% *CI*: 1.30–5.35), diagnosis delay (HR: 2.60; 95% *CI*: 1.42–4.78), CD4^+^ T cell count less than 50/mm^3^ (HR: 2.38; 95% *CI*: 1.27–4.43) and pulmonary atelectasis (HR: 2.20; 95% *CI*: 1.05–4.60), might independently contribute to poor survival. Among patients without cART before anti-TB treatment, the later initiation of cART (more than 8 weeks after starting anti-TB treatment) was found to increase the mortality rate (*OR*: 4.33; 95% *CI*: 1.22–15.36), while the initiation of cART within 4–8 weeks after starting anti-TB treatment was associated with the fewest deaths (0/14).

**Conclusions:**

The subjects in this study conducted in the cART era were still characterized by depressed immunological competence and low rates of cART administration, revealing possible intervention targets for preventing TB reactivation in HIV-infected individuals under current circumstances. Furthermore, our study indicated that the timely diagnosis of PTB, prevention of secondary bacterial pneumonia by prophylactic management and optimization of the timing of cART initiation could have significant impacts on decreasing mortality among HIV/PTB co-infected populations. These findings deserve further prospective investigations to optimize the management of HIV/PTB-co-infected patients.

**Trial registration:**

NCT01344148, Registered September 14, 2010.

**Electronic supplementary material:**

The online version of this article (10.1186/s40249-018-0405-8) contains supplementary material, which is available to authorized users.

## Multilingual abstracts

Please see Additional file 1 for translations of the abstract into the five official working languages of the United Nations.

## Background

Globally, tuberculosis (TB) and human immunodeficiency virus (HIV) co-infection is a major public health problem. It has been estimated that more than one-third of individuals with HIV infection develop active TB infection [1]. Additionally, in 2015, the World Health Organization (WHO) reported that approximately 11% of the 10.4 million incident cases of TB infection worldwide occurred in patients co-infected with HIV [2]. Particularly in developing countries with limited medical resources, TB is one of the most common opportunistic infections in acquired immunodeficiency syndrome (AIDS) patients [3–5].

Both TB and HIV drastically affect the host’s immune system because they can evade immune surveillance and clearance, although the mechanism is not fully understood [6]. In individuals co-infected with HIV and TB, the two pathogens potentiate each other and accelerate the deterioration of immunological functions, resulting in poor outcomes [6]. Compared with that in non-HIV-infected populations, the risk of latent TB activation increases 20-fold in HIV-infected patients [7]. TB infection has also been reported to exacerbate HIV replication [8]. With TB infection, the cytokines induced by immune responses could enhance the replication of HIV and accelerate AIDS progression [9]. For this reason, TB infection has been one of the most common causes of death in HIV-infected patients, accounting for 26% of AIDS-related deaths [7, 10], almost (99%) of which occurred in developing countries [6].

Even in the era of combination antiretroviral therapy (cART), TB infection still places a great burden on people living with HIV (PLWH) in mainland China, a developing country. As revealed in a meta-analysis including 29 studies conducted in mainland China, the overall prevalence of TB infection among PLWH was estimated to be 7.2% (range: 4.2%–12.3%) and was even higher (22.8%) in AIDS patients [11]. In another study conducted among hospitalized patients with HIV infection in China, most AIDS-related deaths were due to TB infection [3]. Under such circumstances, knowledge of the survival and risk factors affecting mortality in HIV-infected patients with PTB would provide important insights for the clinical management of this population, which has yet to be described in the current Chinese context. Hence, we present this study to assess the survival outcome and identify the risk factors for mortality in a retrospective cohort containing 359 HIV-infected subjects with pulmonary tuberculosis (PTB) in Shanghai, China, among whom 49 cases also presented with extra-pulmonary tuberculosis.

## Methods

### Study setting and participants

This study was retrospectively conducted among patients with HIV infection admitted to Shanghai Public Health Clinical Center (SPHCC) for medical care from January 2011 to December 2015. SPHCC is a tertiary hospital affiliated with Fudan University and is the only designated medical institution providing HIV/AIDS care for PLWH in Shanghai. Presently, more than 6000 HIV-infected individuals in Shanghai are being regularly followed up at SPHCC. In 2016, more than 1400 HIV-infected patients were admitted to SPHCC for AIDS or non-AIDS-related illnesses.

The inclusion criteria were as follows: (1) individuals older than 15 years with laboratory-confirmed HIV infection; (2) patients diagnosed with PTB based on the clinical, radiological and laboratory findings; and (3) patients diagnosed with PTB who underwent anti-TB treatment immediately. The diagnosis criteria of PTB cases in this study were in compliance with the national guidelines on the management of TB infection in China [12]. For laboratory-confirmed PTB cases, one of the following three conditions should be met: (1) 2 positive sputum smears by microscopy; (2) 1 positive sputum smear and 1 positive sputum culture; and (3) 1 positive sputum smear with typical features of active TB infection on chest radiological imaging [12]. For the clinical diagnosis of PTB, one of the four following conditions should be met after three negative sputum smears: (1) clinical symptoms, such as cough, hemoptysis and fever with typical features of active TB infection on chest radiological imaging; (2) typical findings of active TB infection on chest radiological imaging, with strongly positive results of the purified protein derivative (PPD) skin test; (3) typical findings of active TB infection on chest radiological imaging and pathological changes of TB infection in extrapulmonary tissues; and (4) suspicious cases were followed up or given diagnostic anti-TB treatment for three weeks, and other pulmonary diseases were excluded [12].

### Study design

This study was a retrospective cohort study. The eligible cases had been followed up regularly since the PTB diagnosis, and the closing date for follow-up was set at August 31, 2016. If the participants were lost to follow-up, which was defined as cases that could not be traced before the closing date, their last visit was considered, and they were further removed from subsequent follow-up. Additionally, cases that were lost to follow-up 1 month after the PTB diagnosis were excluded from the survival analysis.

First, the demographics and clinical features of the enrolled HIV and PTB co-infected patients were observed and compared between subgroups of survival and death cases. Additionally, for the primary interest of this study, the survival outcome was measured in this cohort. Furthermore, the potential risk factors associated with the mortality of HIV-infected patients with PTB were identified in this study, and the survival differences were analyzed between subjects with or without specific risk factors.

The institutional review board of SPHCC approved this study. Considering that this study was retrospective, noninterventional and anonymous, the Ethics Committee of SPHCC authorized this study without written informed consent from the participants.

### Data collection

All data were retrieved by reviewing the medical records in the electronic database of the hospital information system at SPHCC. The standardized data collection tool was utilized to extract demographic characteristics, clinical symptoms, medical comorbidities besides PTB, results of laboratory and radiological examination, timing of the PTB/HIV diagnosis and treatment. The duration of the follow-up was calculated as the time from the PTB diagnosis until the time of death, last visit or close date.

### Statistical analysis

Statistical analysis was performed using SPSS for Windows (version 19.0; IBM Corp, Armonk, NY). The mean (±*SD*), median (interquartile range, IQR) and frequency (%) were used to describe the patients’ characteristics in each group. The Chi-squared test was used to compare categorical variables, and group comparisons of quantitative variables were performed with the parametric (analysis of variance) or nonparametric (Mann-Whitney U test) method depending on the distribution of the variable. The probability of survival after TB diagnosis was estimated by the Kaplan-Meier method. Univariate and multivariate Cox proportional hazards models were used to determine the risk factors affecting mortality by adjusting for confounding factors, in which the hazard ratio (HR) and its 95% confidence interval (*CI*) were estimated, and all factors with statistical significance in univariate analysis were further included in the multivariate analysis. The survival differences between patients with and without specific risk factors were tested by the Kaplan-Meier method and log-rank test. Statistical significance was set at *P* < 0.05 (two-tailed test).

## Results

### Demographic, clinical and laboratory characteristics

A total of 4914 HIV-infected cases were admitted to SPHCC between January 2011 and December 2015, and 359 of these patients who met the inclusion criteria were identified in the database of the hospital information system. The patients’ demographic, clinical and laboratory profiles were described, as shown in Table 1. Most of these patients were male (325/359, 90.53%), and the median age was 39 years (IQR: 31–52 years). At the time of PTB diagnosis, the patients’ median CD4^+^ T cell count was extremely low, at 51 /mm^3^ (IQR: 23–116 /mm^3^), and only a few patients (98/359, 27.30%) were on cART.

**Table 1:** Demographics and clinical characteristics of HIV-infected patients with PTB

	Overall *N* = 359	Survivals *N* = 280	Deaths *N* = 53	*P* value
Age, Years, median (IQR)	39 (31,52)	39 (29,48)	49 (35,63)	< 0.001
Male gender *N* (%)	325 (90.53%)	254 (90.71%)	48 (90.57%)	1.000
On cART *N* (%)	98 (27.30%)	83 (29.64%)	8 (15.09%)	0.029
Diagnostic delay^a^ *N* (%)	153 (42.60%)	106 (37.86%)	36 (67.92%)	< 0.001
Extrapulmonary Involvement	49 (13.65%)	35 (12.50%)	12 (22.64%)	0.052
Clinical symptoms				
Fever (> 39 °C) *N* (%)	263 (73.26%)	207 (73.93%)	32 (60.38%)	0.044
Cough *N* (%)	181 (50.42%)	144 (51.43%)	28 (52.83%)	0.851
Expectoration *N* (%)	82 (22.84%)	61 (21.79%)	17 (32.08%)	0.105
Anorexia *N* (%)	81 (22.56%)	60 (21.45%)	13 (24.53%)	0.617
Dyspnea *N* (%)	69 (19.22%)	49 (17.50%)	16 (30.19%)	0.033
Complications				
Bacterial pneumonia *N* (%)	32 (8.91%)	18 (6.43%)	14 (26.42%)	< 0.001
HBV/HCV co-infection *N* (%)	25 (6.96%)	19 (6.79%)	6 (11.32%)	0.387
Syphilis *N* (%)	33 (9.19%)	30 (10.71%)	1 (1.89%)	0.077
Diabetes *N* (%)	14 (3.90%)	11 (3.93%)	2 (3.77%)	1.000
Hypertension *N* (%)	12 (3.345%)	9 (3.21%)	2 (3.77%)	1.000
Laboratory examination				
Sputum smear positive *N* (%)	156 (43.45%)	126 (45.00%)	21 (39.62%)	0.470
Sputum culture positive *N* (%)	180 (50.14%)	141 (50.36%)	23 (43.40%)	0.353
T-spot. TB test positive *N* (%)	227 (63.23%)	181 (64.64%)	30 (56.60%)	0.265
HIV RNA lg copies/ml median (IQR)	5.17 (3.91,5.66)	5.14 (3.83,5.65)	5.32 (4.61,5.79)	0.354
CD4^+^ T cell/mm^3^ median (IQR)	51 (23 116)	55 (27 122)	24.5 (10,86)	0.001
Manifestation of lung CT scan				
Extensive pulmonary lesion^b^ *N* (%)	218 (60.72%)	160 (57.14%)	41 (77.36%)	0.006
Pulmonary cavity *N* (%)	36 (10.03%)	25 (8.93%)	9 (16.98%)	0.076
Pulmonary atelectasis *N* (%)	31 (8.64%)	20 (7.14%)	9 (16.98%)	0.039

^a^Diagnostic delay was defined as the pulmonary TB was diagnosed in more than four weeks after the onset of symptoms

^b^Extensive pulmonary lesion was defined as TB lesions involved more than 3 pulmonary lobes by chest CT scan

### Survival analysis

**Fig. 1:**
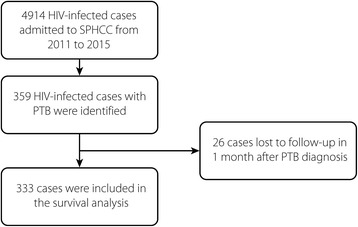
Flow chart showing study subject identification

**Fig. 2:**
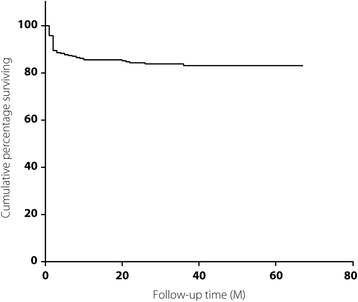
Cumulative survival rate among HIV-infected patients with PTB

Although 26 patients were lost to follow-up 1 month after the diagnosis of PTB, 333 cases were included in the survival analysis, with a median follow-up duration of 27 months (IQR: 13–43 months) (Fig. 1), and this included 9 cases that were lost to follow-up and excluded before the closing date (median follow-up duration: 28 months; IQR: 12–36 months). During the follow-up period, 53 patients died, with an overall mortality rate of 15.92%. The cumulative survival rates at 6, 12 and 24 months were 87.39%, 85.56% and 84.31%, respectively, as shown in Fig. 2, and most deaths occurred within 6 months after the PTB diagnosis (41/53, 77.36%). The clinical and demographic profiles were compared between the death and survival cases. The death cases were characterized by older age (*P* < 0.001), a lower CD4^+^ T cell count (*P* < 0.001), a lower rate of cART administration (*P* = 0.029), being more prone to diagnosis delay (*P* < 0.001), complication with bacterial pneumonia (*P* < 0.001) and clinical manifestations of dyspnea (*P* = 0.033), a reduced presence of high fever (*P* = 0.044), more extensive lesions (*P* = 0.006) and pulmonary atelectasis (*P* = 0.039) shown on chest CT (Table 1).

### Risk factors affecting survival outcome

**Fig. 3:**
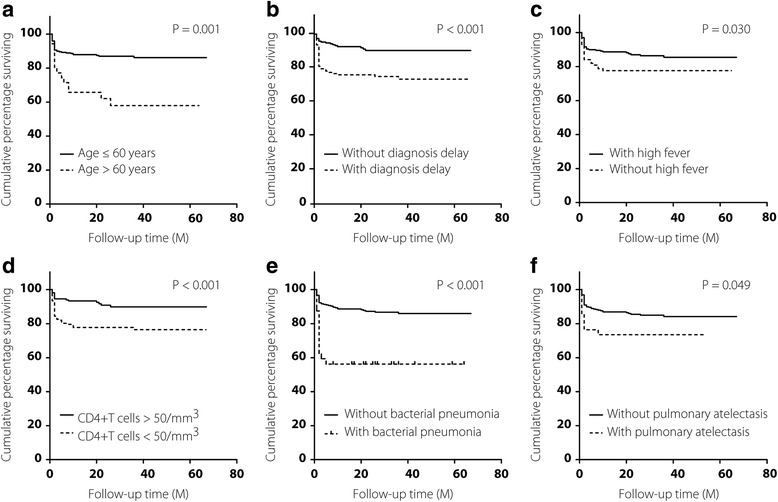
Kaplan-Meier curves comparing the outcomes affected by risk factors (**a**, age > 60 years; **b**, diagnosis delay; **c**, high fever; **d**, CD4^+^ T cell count < 50 /mm^3^; **e**, bacterial pneumonia; **f**, pulmonary atelectasis)

The variables listed in Table 2 were included in the Cox proportional hazards model to assess potential risk factors for mortality. After conducting univariate (UV) and multivariable (MV) analyses, older age (> 60 years) (UV: HR: 3.31, 95% *CI*: 1.80–6.10; MV: HR: 3.18, 95% *CI*: 1.66–6.10), a diagnosis delay (UV: HR: 3.05, 95% *CI*: 1.71–5.44; MV: HR: 2.60, 95% *CI*: 1.42–4.78), the absence of high fever (UV: HR: 0.56; 95% *CI*: 0.32–0.97; MV: HR: 0.54, 95% *CI*: 0.30–0.96), complication with bacterial pneumonia (UV: HR: 4.07, 95% *CI*: 2.21–7.52; MV: HR: 2.64, 95% *CI*: 1.30–5.35), a CD4^+^ T cell count less than 50 /mm^3^ (UV: HR: 2.70, 95% *CI*: 1.48–4.90; MV: HR: 2.38, 95% *CI*: 1.27–4.43) and pulmonary atelectasis (UV: HR: 2.29, 95% *CI*: 1.12–4.70; MV: HR: 2.20, 95% *CI*: 1.05–4.60) shown on chest CT scan were identified as independent risk factors contributing to poor survival in these patients (Table 2). As depicted by Kaplan-Meier curves and tested by the log-rank tests, all these factors were significantly associated with decreased cumulative survival rates (Fig. 3).

**Table 2:** Risk factors associated with the mortality of HIV infected patients with PTB^a^

Risk factor	Univariate analysis		Multivariate analysis	
	HR^b^ (95% *CI*)	*P* value	HR^b^ (95% *CI*)	*P* value
Age > 60	3.31 (1.80–6.10)	< 0.001	3.18 (1.66–6.10)	0.001
Male gender	0.97 (0.39–2.43)	0.942		
Not on cART	2.17 (1.03–4.61)	0.043	1.48 (0.67–3.25)	0.332
Diagnosis delay	3.05 (1.71–5.44)	< 0.001	2.60 (1.42–4.78)	0.002
Clinical symptoms				
Fever (> 39 °C)	0.56 (0.32–0.97)	0.037	0.54 (0.30–0.96)	0.037
Cough	1.05 (0.61–1.81)	0.850		
Expectoration	1.58 (0.89–2.81)	0.122		
Anorexia	1.14 (0.61–2.12)	0.690		
Dyspnea	1.88 (1.04–3.37)	0.036	1.03 (0.53–1.97)	0.941
Extrapulmonary Involvement	1.83 (0.96–3.48)	0.066		
CD4^+^ T cell count < 50 cells/mm^3^	2.70 (1.48–4.90)	0.001	2.38 (1.27–4.43)	0.007
Comorbidities				
Bacterial pneumonia	4.07 (2.21–7.52)	< 0.001	2.64 (1.30–5.35)	0.007
HBV/HCV co-infection	1.57 (0.67–3.68)	0.298		
Syphilis	0.18 (0.03–1.32)	0.093		
Diabetes	0.96 (0.23–3.92)	0.949		
Hypertension	1.13 (0.27–4.62)	0.871		
Manifestation of lung CT scan				
Extensive pulmonary lesion	2.39 (1.26–4.55)	0.008	1.76 (0.90–3.44)	0.100
Pulmonary cavity	2.00 (0.98–4.10)	0.058		
Pulmonary atelectasis	2.29 (1.12–4.70)	0.023	2.20 (1.05–4.60)	0.037

^a^Statistical analysis was performed by the Cox proportional-hazards model

^b^HR indicated relative mortality risk for cases with the specific risk factor referred to cases without the corresponding risk factor

**Fig. 4:**
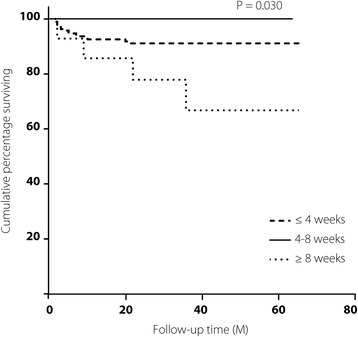
Kaplan-Meier curves comparing the outcomes affected by the timing of cART initiation after starting anti-TB treatment

Among the 217 patients who initiated cART after starting anti-TB treatment, the timing of cART initiation was associated with the survival outcome (*χ*^2^ = 7.03, *P* = 0.030), as shown in Fig. 4. Compared with patients who initiated cART within 4 weeks after beginning anti-TB treatment (Table 3), those who began cART later (more than 8 weeks after starting anti-TB treatment) had a worse survival outcome (*OR*: 4.33, 95% *CI*: 1.22–15.36). Additionally, we found that the initiation of cART within 4–8 weeks after starting anti-TB treatment was associated with the fewest deaths (Table 3), but the difference did not reach statistical significance compared with patients initiating cART within 4 weeks after starting anti-TB treatment (*P* = 0.257).

**Table 3:** Survival differences among patients with different timing of cART initiation after staring anti-TB treatment^a^

Timing of cART initiation since starting anti-TB treatment	Mortality rate	OR (95% *CI*)	*P* value
≤4 weeks	8.47% (16/189)	1	
4–8 weeks	0% (0/14)	NA^b^	0.257
≥8 weeks	28.57% (4/14)	4.33 (1.22–15.36)	0.015

^a^Statistical analysis was performed by the chi-square test

^b^Due to the value of variable is zero, *OR* could not be calculated

## Discussion

In China and other developing countries, TB is the most common and life-threatening opportunistic infection in HIV-infected patients [3, 4, 13]. Our study revealed the clinical features and survival outcomes of patients co-infected with HIV and PTB. Furthermore, the potential risk factors impacting mortality were evaluated. We also found that the timing of cART initiation could affect the survival outcome in cART-naïve patients undergoing anti-TB treatment.

In this study, most of the HIV-infected patients with PTB presented with severe immunodeficiency (CD4^+^ T cell count < 50 /mm^3^). Additionally, at the time of the PTB diagnosis, fewer than 30% of patients in this cohort had been on cART. These findings correspond with previously reported data demonstrating the close relationship between TB activation and the immunosuppressive state of the host [4, 14–16]. As in the general population, most individuals infected with *Mycobacterium tuberculosis* latently do not develop active TB infection, but individuals co-infected with HIV do [17]. HIV-induced immunosuppression presenting as the loss of CD4^+^ T cells in the host’s peripheral blood, lymphoid tissues, and mucosa has been regarded as the most significant risk factor for primary infection and reactivation of TB [17]. Additionally, in a meta-analysis, cART administration was strongly associated with a reduced risk of TB activation [18], indicating that cART could be the most important measure to control TB activation in HIV-infected individuals by restoring the host’s immune function [19]. Hence, our findings imply that enforcing the timely initiation of cART is required to reduce the TB burden among HIV-infected populations in the current context in China.

During the median 26 months of follow-up, the overall survival rate in this cohort was comparable to that reported in recent studies of HIV/TB co-infected patients from other developing countries [4, 20–22]. While this study was conducted during the cART era, an advanced immunosuppression state and lower rate of cART administration were still identified as characteristics of death case. Furthermore, the host’s CD4^+^ T cell count had an independent impact on the prognosis of AIDS patients with PTB. Additionally, in other studies, the introduction of cART could drastically reduce the mortality of HIV and TB co-infected patients by effectively suppressing the replication of HIV and concomitantly raising the peripheral CD4^+^ T cell count [22–24]. As recommended in the WHO management guideline for HIV and TB co-infection, cART should be administered as soon as possible after the initiation of anti-TB treatment (within 8 weeks) without consideration of the CD4^+^ T cell count [25]. Consistently, in the survival analysis for patients who initiated cART after starting anti-TB treatment, our findings revealed that the later initiation of cART (more than 8 weeks after starting anti-TB treatment) could increase the risk of mortality. However, our analysis also indicated that the death rate among patients who initiated cART within 4–8 weeks after starting anti-TB treatment was lower than that in patients initiating cART within 4 weeks, although this difference did not reach statistical significance. Considering that most patients in this cohort showed an extremely low profile of the CD4^+^ T cell count when diagnosed with PTB, rapid CD4^+^ T cell restoration induced by cART could lead to the phenomenon known as immune reconstitution inflammatory syndrome (IRIS), which could affect the prognosis of HIV/TB co-infection patients [17, 23]. Based on previous findings, it was suggested that the initiation of cART should be delayed in HIV and TB co-infected patients with CD4^+^ T cell counts < 100 cells/mm^3^ [23, 26, 27]. Additionally, for HIV and TB co-infected patients with CD4 T-cell counts > 200 cells/mm^3^, the most recent evidence even supported that cART initiation could be delayed until completion of the 6 months of anti-TB treatment to avoid adverse events [28]. While the optimized timing of cART initiation after starting anti-TB therapy has not been determined, the results of our study indicate that the initiation of cART within 4–8 weeks after the start of anti-TB treatment could improve the survival outcome, especially for patients with low CD4^+^ T cell counts. The findings deserve further prospective investigations in the future.

The early diagnosis and immediate initiation of anti-TB treatment are essential for TB management, while HIV infection has been identified as an important factor associated with a delayed diagnosis of TB infection in a systemic review [29]. Consistently, in this study, a diagnosis delay was common, as more than 40% subjects were diagnosed with PTB later than 4 weeks after disease onset. Several reasons lead to the delay of the PTB diagnosis in HIV-infected patients, including extensive involvement of the lower lung lobes, atypical chest radiographic findings, low concentrations of bacteria in the sputum and frequent extrapulmonary involvement [30]. The role of delay in the diagnosis of TB is significant for both disease progression and patient outcome [29] and has been reconfirmed in our study among HIV/PTB co-infected individuals. Currently, there are numerous novel technologies to improve the diagnosis of TB in patients with HIV infection. In a randomized trial conducted in areas with a high prevalence of TB and HIV infection, use of the Xpert method to diagnose TB infection in primary care clinics increased the number of confirmed TB cases and reduced the time to treatment initiation among HIV-infected patients [31]. Future studies to investigate the role of these newly developed TB diagnostic methods to improve the outcome of AIDS patients with PTB are expected.

Similar to that reported in previous studies conducted in HIV- or non-HIV-infected patients with PTB, the prognosis was worse among elderly patients (≥60 years old) than among younger individuals in this cohort, possibly attributed to a lower body weight, coexisting medical diseases and extensive radiographic involvement in elderly patients [21, 32]. Additionally, other risk factors, such as the absence of high fever, manifestation of dyspnea, complication of bacterial pneumonia and pulmonary atelectasis shown on chest CT scan, were found to be associated with poor survival in this cohort. Future studies to optimize the management of these clinical challenges in HIV/TB co-infection patients should be conducted.

Admittedly, several limitations were endowed in this study. First, it was an observational and retrospective study; recall bias from medical record review could not be eliminated. Meanwhile, this study did not include non-HIV-infected patients with PTB as a proper control group, which could strengthen the conclusions of our study. Additionally, the cases lost to follow-up in this cohort could be a bias to more precisely estimate the survival outcome. We also note that the findings revealed in this study do not fully represent the overall view of the outcome for HIV and PTB co-infected patients of China, especially in other regions with different socioeconomic and medical resource statuses. Finally, while the survival outcomes and risk factors affecting mortality identified in this study provide insight into improving clinical management and promoting further investigations of the HIV/PTB co-infection population in China, it is important to note that these findings need to be reappraised, especially in future interventional or prospective observational studies.

## Conclusions

This study revealed the survival outcome of HIV-infected patients with PTB in China, which was comparable to that reported in other developing regions. Currently, HIV-infected patients complicated with PTB in China are still characterized by a depressed immunological profile and low rates of cART administration, indicating that promoting the timely administration of and adherence to cART remain the primary measures to reduce the TB burden in HIV-infected subjects under such circumstances. Additionally, for clinical management, our findings identified that several factors, including older age, a diagnosis delay, complication with bacterial pneumonia, serious disease manifestations and the timing of cART initiation, could be challenges to improving the survival outcome in HIV/PTB co-infected populations. To explore optimization strategies for HIV/PTB management, further investigations based upon these findings should be conducted.

## Additional file

Additional file 1: Multilingual abstracts in the five official working languages of the United Nations. (PDF 210 kb)

## Abbreviations

AIDS: Acquired immunodeficiency syndrome
cART: Combination antiretroviral therapy
*CI*: Confidence interval
HIV: Human immunodeficiency virus
HR: Hazard ratio
IQR: Interquartile range
*OR*: Odds ratio
PLWH: People living with HIV infection
PTB: Pulmonary tuberculosis
SPHCC: Shanghai Public Health Clinical Center
TB: Tuberculosis
WHO: World Health Organization
