# Supplementary material for: Risk factors affecting the mortality of HIV-infected patients with pulmonary tuberculosis in the cART era: a retrospective cohort study in China
Source: Infect Dis Poverty. 2018 Mar 24;7:25. doi: 10.1186/s40249-018-0405-8 (PMC5870507; doi:10.1186/s40249-018-0405-8)
Supplement: Supplementary file 1 — Multilingual abstracts in the five official working languages of the United Nations. (PDF 210 kb) [file 40249_2018_405_MOESM1_ESM.pdf]

Translation of the abstract into the five official working languages of the United Nations

عوامل الخطر التي تؤثر على وفيات المرضى المصابين بعدوى فيروس نقص المناعة البشرية مع السل الرئويفي زمن العلاج المركب المضاد للفيروسات القهقرية: دراسة أترابية استرجاعية في الصين

يونغ-جيا جي، باي-باي ليانغ، جيا-يِن شِن، جيان-جُن سُن، جُن-يانغ يانغ، جُن تشن، تانغ-كاي كي، تشن-يان وانغ، واي سونغ، يانغ تانغ، لي ليو، رِن-فانغ تشانغ، يِن-تشونغ شِن، هونغ تشو لو

الملخص
خلفية: لا تزال عدوى السل تشكل عبئًا كبيرًا على الأفراد المصابين بفيروس نقص المناعة البشرية في الصين ودول نامية أخرى. ومن شأن المعرفة في مسألة نجاة المرضى المصابين بفيروس نقص المناعة البشرية مع السل الرئوي أن توفر رؤى هامة للإدارة السريرية لهذه الفئة من السكان، والتي لا تزال يرد وصفها بدقة في الصين حاليًا.
الأساليب: سُجل المرضى المصابين بفيروس نقص المناعة البشرية مع السل الرئوي الذين تم إدخالهم إلى مركز شنغهاي للصحة السريرية العامة بين يناير 2011 وحتى ديسمبر 2015 تسجيلاً استرجاعيًا . ففي الدراسة الأترابية هذه، قُدّر حجم التنبؤبالنجاةعلى طريقة كابلان-ماير، في حين استخدمت نماذج كوكس للمخاطر النسبية أحادية ومتعددة المتغيرات لتحديد عوامل الخطر المؤثرة على الوفيات.
النتائج: <bx/>بعد مراجعة 4914 حالة لمرضى مدخلين مصابين بفيروس نقص المناعة البشرية، تم التعرف على 359 حالة مصابة بالسل الرئوي. وأثناء تشخيص حالات السل الرئوي، كان متوسط عدد خلايا CD4+ T 51/مم^{3} (IQR: 23-116)، وبلغت نسبة المرضى الذين يحصلون على العلاج المركب المضاد للفيروسات القهقرية 27.30% (98/359). وبلغ المعدل الكلي للوفيات في الـ333 حالة المشمولة في تحليل النجاة 15.92% (53/333) خلال متوسط 27 شهرًا من المتابعة. ومن الممكن أن تساهم عوامل الخطر التي تشمل أن يزيد العمر عن 60 عامًا (HR: 3.18؛ 95% CI: 1.66-6.10)، فضلاً عن المضاعفات مع الالتهاب الرئوي الجرثومي (HR: 2,64؛ 95% *CI*: 1.30-5.35)، والتأخر في التشخيص (HR: 2,60؛ 95% *CI*: 1.42 – 4.78)، وأن يكون عدد خلايا CD4+ T أقل من 50/مم ^{3} (HR: 2.38; 95% *CI*: 1.27 – 4.43)، والانخماص الرئوي (HR: 2.20; 95% *CI*: 1.05 – 4.60), في ضعف إمكانية النجاة. ومن بين المرضى الذين لم يتناولوا العلاج المركب المضاد للفيروسات القهقرية قبل العلاج المضاد للسل الرئوي، تبين أن بدء العلاج المركب المضاد للفيروسات القهقرية في مرحلة لاحقة (أكثر من 8 أسابيع بعد بدء العلاج المضاد للسل) يزيد من معدل الوفيات (*OR*: 4.33; 95% *CI*: 1.22 – 15.36)، بينما ارتبط بدء العلاج المركب بالفيروسات القهقرية في غضون 4-8 أسابيع بعد بدء العلاج المضاد للسل بعدد أقل من الوفيات (0/14).
الاستنتاجات: لا تزال الموضوعات في هذه الدراسة التي أجريت في عصر العلاج المركب المضاد للفيروسات القهقرية توسم بانخفاض الكفاءة المناعية وانخفاض معدلات إعطاء هذا النوع من العلاج، مما يكشف عن أهداف التدخل المحتملة لمنع إعادة تنشيط السل لدى الأفراد المصابين بفيروس نقص المناعة البشرية في ظل الظروف الراهنة. علاوة على ذلك، تشير دراستنا إلى أن التشخيص في الوقت المناسب للسل، والوقاية من الالتهاب الرئوي الجرثومي عبر الإدارة الوقائية والاستفادة المثلى من توقيت بدء العلاج المركب المضاد للفيروسات القهقرية يمكن أن يكون له آثار كبيرة على تقليل الوفيات بين المصابين بفيروس نقص المناعة البشرية/السل في الوقت ذاته. وتستحق هذه النتائج المزيد من التحقيقات المستقبلية لتحسين إدارة المرضى المصابين بفيروس نقص المناعة البشرية/السل الرئوي في الوقت ذاته.

Translated from English version into Arabic by Shada Salameh and Mahmoud Sami, through

TRANSLATORS
WITHOUT BORDERS

HIV 感染合并肺结核患者死亡相关危险因素分析：一项在中国开展的回顾性队列研究

纪永佳，梁佩佩、沈佳胤、孙建军、杨君洋、陈军、齐唐凯、王珍燕、宋玮、汤阳、刘莉、张仁芳、沈银忠、卢洪洲

摘要：

研究背景：对于中国及其他发展中国家 HIV 感染者来说，结核感染相关疾病负担依旧沉重。现今中国 HIV 感染合并肺结核患者的生存状况仍有待研究，而相关研究结论对于优化该人群的临床治疗可提供重要依据。

研究方法：本研究纳入 2011.1 至 2015.12 期间就诊于上海市公共卫生临床中心的 HIV 感染合并肺结核患者进行回顾性分析，通过 Kaplan-Meier 方法估算该队列生存预后，同时运用 Cox 比例风险模型进行单因素及多因素分析死亡相关危险因素。

研究结果：通过回顾 4914 名 HIV 感染住院患者的病历资料，359 名符合诊断标准的肺结核患者被纳入研究。当被确诊为肺结核时，患者中位 CD4+ T 细胞计数为 51/mm^3 （*IQR*: 23-116），其中 27.30%患者（98/359）正在接受联合抗逆转录病毒治疗（cART）。被纳入生存分析研究 333 名患者中，中位 27 个月随访期间总体病死率为 15.92%（53/333）。进一步分析发现，HIV 感染合并肺结核患者死亡相关独立风险因素包括：年龄大于 60 岁（*HR*: 3.18; 95% *CI*: 1.66-6.10）、并发细菌性肺炎（*HR*: 2.64; 95% *CI*: 1.30 – 5.35）、结核诊断延迟 （*HR*: 2.60; 95% *CI*: 1.42 – 4.78）、CD4+ T 细胞计数小于 50/mm^3 （*HR*: 2.38; 95% *CI*: 1.27 – 4.43）以及肺不张（*HR*: 2.20; 95% *CI*: 1.05 – 4.60）。对于抗结核治疗开始前未接受 cART 的患者来说，延迟启动 cART（抗结核治疗开始 8 周后）患者死亡风险升高（*OR*: 4.33; 95% *CI*: 1.22 – 15.36），而抗结核治疗开始 4-8 周内启动 cART 的患者死亡发生率最低（0/14）。

研究结论：尽管 HIV 治疗已进入 cART 时代，本研究发现免疫功能严重缺陷和 cART 治疗率低下仍是中国 HIV 感染合并肺结核患者重要特征，该发现对降低 HIV 感染者人群中结核感染激活发生提供了干预目标。此外根据研究结果，及时诊断肺结核、预防继发性肺部细菌感染和优化 cART 治疗时机等可能是降低 HIV 感染合并肺结核患者死亡风险的重要措施。将来在本研究基础之上，需要开展前瞻性研究进一步评估上述因素对于 HIV 感染合并肺结核患者预后的影响，为优化该人群的诊断和治疗提供依据。

Translated from English version into Chinese by Yong-Jia Ji

Facteurs de risque influant sur la mortalité des patients infectés par le VIH et atteints de tuberculose pulmonaire à l’âge du TARc : étude de cohorte rétrospective en Chine

Yong-Jia Ji, Pei-Pei Liang, Jia-Yin Shen, Jian-Jun Sun, Jun-Yang Yang, Jun Chen, Tang-Kai Qi, Zhen-Yan Wang, Wei Song, Yang Tang, Li Liu, Ren-Fang Zhang, Yin-Zhong Shen, Hong-Zhou Lu

Résumé

Contexte : Les infections tuberculeuses constituent toujours un lourd fardeau pour les patients infectés par le VIH en Chine et dans d'autres pays en voie de développement. La détermination de la survie des patients infectés par le VIH et atteints de tuberculose pulmonaire apporterait de précieuses informations pour la prise en charge clinique de cette population, encore mal décrite en Chine à ce jour.

Méthodes : Des patients infectés par le VIH et atteints de tuberculose, admis au Centre clinique de santé publique de Shanghai entre janvier 2011 et décembre 2015, ont été enrôlés rétrospectivement. Dans cette cohorte, le pronostic de survie a été estimé par la méthode de Kaplan-Meier, tandis que des modèles proportionnels de Cox uni- et multivariés étaient utilisés pour déterminer les facteurs de risque influant sur la mortalité.

Résultats : La revue de 4914 patients séropositifs hospitalisés a permis d'identifier 359 cas de tuberculose pulmonaire. Au moment du diagnostic de tuberculose, la numération de CD4+ médiane de ces patients était de 51/mm^3 (EI de 23 à 116) et 27,30 % des patients (98 sur 359) étaient sous traitement antirétroviral combiné (TARc). Sur les 333 cas inclus dans l'analyse de survie, la mortalité globale était de 15,92 % (53 sur 333) avec un recul médian de 27 mois. Les facteurs de risque, notamment un âge supérieur à 60 ans (HR : 3,18 ; IC à 95 % de 1,66 à 6,10), des complications par une pneumonie bactérienne (HR : 2,64 ; IC à 95 % de 1,30 à 5,35), un diagnostic tardif (HR : 2,60 ; IC à 95 % de 1,42 à 4,78), une numération de CD4+ inférieure à 50/mm^3 (HR : 2,38 ; IC à 95 % de 1,27 à 4,43) et une atélectasie pulmonaire (HR : 2,20 ; IC à 95 % de 1,05 à 4,60), pourraient contribuer indépendamment à un faible taux de survie. Parmi les patients sous TARc avant d'être traités contre la tuberculose, la mise en place tardive du TARc (plus de 8 semaines après le début du traitement antituberculeux) s'avère augmenter le taux de mortalité (OR : 4,43 ; IC à 95 % de 1,22 à 15,36), tandis que sa mise en place dans les 4 à 8 semaines après le début du traitement antituberculeux est associée au plus faible taux de mortalité (0 sur 14).

Conclusions : Les sujets de l'étude menée à l'époque du TARc étaient encore caractérisés par une immunodépression et un faible taux d'administration du TARc, ce qui révèle des cibles possibles d'intervention pour la prévention de la réactivation de la tuberculose chez les sujets infectés par le VIH dans les circonstances actuelles. En outre, notre étude montre que le diagnostic précoce de la tuberculose pulmonaire, la prévention des pneumonies bactériennes secondaires et l'optimisation du moment de mise en place du TARc pouvaient contribuer significativement à réduire la mortalité dans les populations co-infectées par le VIH et la tuberculose pulmonaire. Ces observations méritent que l'on y revienne dans le cadre d'études prospectives, afin d'optimiser la prise en charger des patients co-infectés par le VIH et la tuberculose pulmonaire.

Translated from English version into French by Suzanne Assenat, through

TRANSLATORS
WITHOUT BORDERS

Факторы риска, влияющие на смертность ВИЧ пациентов с лёгочным туберкулёзом в эру КАРВТ: ретроспективное когортное исследование в Китае

Юн-Цзя Цзи, Пэй-Пэй Лян, Цзя-Инь Шэнь, Цзянь-Цзюнь Сунь, Цзюнь-Ян Ян, Цзюнь Чэнь, Тан-Кай Ки, Чжэнь-Янь Ван, Вэй Сон, Ян Тан, Ли Лю, Жэнь-Фан Чжан, Инь-Чжон Шэнь, Хун-Чжоу Лу

Аннотация

Краткая информация: Инфекция туберкулёза всё ещё является тяжёлым бременем для ВИЧ-инфицированных пациентов в Китае и других развивающихся странах. Исследования по выживаемости ВИЧ-инфицированных пациентов с лёгочным туберкулёзом (ЛТБ) обеспечат важной информацией по клиническому ведению групп населения, которые нуждаются в освещении в Китае в настоящее время.
Методы: Были ретроспективно отобраны ВИЧ-инфицированные пациенты с ЛТБ, принятые в Шанхайский Центр общественного здравоохранения с января 2011 г. по декабрь 2015 г. В этой когорте, прогноз выживаемости был рассчитан по методу Каплана-Майера, в то время как для определения факторов риска, влияющих на смертность, использовались одномерные и многомерные модели пропорциональной опасности Кокса.
Результаты: После обзора 4 914 ВИЧ-инфицированных пациентов было выявлено 359 случаев ЛТБ. Во время диагностики ЛТБ средний показатель количества клеток CD4 + T пациентов составлял 51 / мм^3 (IQR: 23-116), и 27,30% пациентов (98/359) были на комбинированной антиретровирусной терапии (КАРВТ). По 333 случаям, включённым в анализ выживаемости, общая смертность составила 15,92% (53/333) за период среднего 27-месячного наблюдения. Факторы риска, включая возраст старше 60 лет (HR: 3,18; 95%CI: 1,66-6,10), осложнение бактериальной пневмонией (HR: 2,64; 95% *CI*: 1,30 – 5,35), задержку диагностики (HR: 2.60; 95% *CI*: 1,42 – 4,78), количество лимфоцитов CD4 + менее 50 мм^3 (HR: 2,38; 95% *CI*: 1,27 – 4,43) и ателектаз легких (HR: 2,20; 95% *CI*: 1,05 – 4,60), может, в частности, послужить причиной плохой выживаемости. Было обнаружено, что среди пациентов, не принимавших КАРВТ до начала лечения ТБ, последующее начало АРВТ (более 8 недель после начала противотуберкулезного лечения) увеличивало смертность(*OR*: 4,33; 95% *CI*: 1,22 – 15,36), в то время как инициция КАРВТ в течение 4-8 недель после начала противотуберкулёзного лечения была связана с меньшей смертностью (0/14).
Выводы: Субъекты этого исследования, которое проводилось во время АРВТ, по-прежнему характеризуются пониженной иммунологической компетентностью и низким уровнем введения КАРВТ, что является возможной целью по предотвращению возобновления ТБ у ВИЧ-инфицированных пациентов при данных обстоятельствах. Кроме того, наше исследование продемонстрировало, что своевременная диагностика ЛТБ, и предотвращение вторичной бактериальной пневмонии с помощью профилактических мер, а также оптимизации сроков КАРВТможет иметь значительное влияние на снижение смертности среди групп пациентов, ко-инфицированных ВИЧ/ЛТБ. Эти результаты требуют дальнейшего перспективного исследования для оптимизации лечения пациентов, одновременно инфицированных ВИЧ и ЛТБ.

Translated from English version into Russian by Liudmila Tomanek and Gulnoza Ahmedova, through

TRANSLATORS
WITHOUT BORDERS

Factores de riesgo que afectan la mortalidad de pacientes infectados de VIH con tuberculosis

pulmonar en la era del TARc: un estudio retrospectivo de cohorte en China

Yong-Jia Ji, Pei-Pei Liang, Jia-Yin Shen, Jian-Jun Sun, Jun-Yang Yang, Jun Chen, Tang-Kai Qi, Zhen-Yan Wang, Wei Song, Yang Tang, Li Liu, Ren-Fang Zhang, Yin-Zhong Shen, Hong-Zhou Lu

Resumen

Antecedentes: La infección por tuberculosis aún supone una gran carga para las personas infectadas de VIH en China y en otros países en desarrollo. El conocimiento de la supervivencia de pacientes infectados de VIH con tuberculosis pulmonar (TBP) sería una gran ayuda parala gestión clínica de estapoblación, de la que aún queda mucho por saber en la actual China.
Métodos: Los pacientes infectados de VIH con TBP ingresados en el Centro Clínico de Salud Pública de Shanghai desde enero de 2011 hasta diciembre de 2015 se inscribieron retrospectivamente. En esta cohorte, se calculó el pronóstico de supervivencia mediante el método Kaplan-Meier, en tanto que se utilizaron los modelos de riesgo proporcional univariantes y multivariantes de Cox para determinar los factores de riesgo que afectan la mortalidad.
Resultados: Tras revisar a los 4.914 pacientes ingresados infectados con VIH, se identificaron 359 casos de TBP. En el momento del diagnóstico de TBP, el recuento medio de células de pacientes CD4+ T era de 51/mm^3 (IQR: 23-116) y el 27,30% de pacientes (98/359) seguían un tratamiento antirretroviral combinado (TARc). En los 333 casos incluidos en el análisis de supervivencia, la mortalidad total fue de 15,92% (53/333) durante un seguimiento medio de 27 meses. Los factores de riesgo, incluyendo edad superior a los 60 años (HR: 3,18; 95%CI: 1,66-6,10), complicaciones con neumonía bacteriana (HR: 2,64; 95% *CI*: 1,30 – 5,35), retraso en el diagnóstico (HR: 2,60; 95% *CI*: 1,42 – 4,78), recuento de células CD4+ T menor de 50/mm^3 (HR: 2,38; 95% *CI*: 1,27 – 4,43) y atelectasia pulmonar (HR: 2,20; 95% *CI*: 1,05 – 4,60), podrían contribuir de manera independiente a una supervivencia reducida. Entre los pacientes sin TARc anterior al tratamiento anti TB, se descubrió que un inicio tardío del TARc (más de 8 semanas tras comenzar con el tratamiento anti TB) aumentaba la tasa de mortalidad (*O*: 4,33; 95% *CI*: 1,22 – 15,36), mientras que el inicio de TARc dentro de las 4 – 8 semanas tras comenzar con el tratamientoanti TB se asoció con menos muertes (0/14).
Conclusiones: Los sujetos de este estudio realizado en la era del TARc se caracterizaban por una competencia inmunológica deprimida y una baja tasa de administración de TARc, lo que muestra que podría haber objetivos en los que intervenir para prevenir la reactivación de la TB en las personas infectadas de VIH en las circunstancias actuales. Además, nuestro estudio indicó que el diagnóstico a tiempo de TBP, la prevención de la neumonía bacteriana secundaria mediante la gestión profiláctica y la optimización en los tiempos de iniciación del TARc podrían tener impactos significativos en el descenso de la mortalidad entre las poblaciones de pacientes infectados de VIH y TBP. Estos hallazgos médicos merecen más investigaciones futuras a fin de optimizar la gestión de pacientes infectados simultáneamente de VIH y TBP.

Translated from English version into Spanish by Carla Salvador Tato, through

TRANSLATORS
WITHOUT BORDERS
